# Design of Web-to-Web Spacing for the Reduced Pressure Drop and Effective Depth Filtration

**DOI:** 10.3390/polym11111822

**Published:** 2019-11-06

**Authors:** Sanghyun Roh, Kangsoo Park, Jooyoun Kim

**Affiliations:** 1 Department of Textiles, Merchandising and Fashion Design, Seoul National University, Seoul 08826, Korea; 2 R & D Center, Satrec Initiative Co., Ltd., Daejeon 34054, Korea; mepgsoo@gmail.com; 3 Research Institute of Human Ecology, Seoul National University, Seoul 08826, Korea

**Keywords:** layer construction, spacer web, air gap, pressure drop, service life, depth filtration

## Abstract

The study aims at lowering the pressure drop and extending the service life at a given set of filter materials implementing a space between the filter layers. As design factors, the web-to-web space was implemented by inserting either a bulk air gap or porous spacer web between the filter webs. The effect of spacing, either by the air gap or by the spacer web, on the pressure drop reduction was apparent for 4-layer constructions, and the effect was greater at the higher face velocity. The use of spacer web was more effective than the air gap in reducing the pressure drop, because the porous, fluffy spacer web acted as an effective air flow channel between the compact filter layers. The loading capacity was also increased with the spacer web implementation, effectively delaying the clogging point and extending the service life. Employing both experimental investigation and numerical simulation, this study intended to provide a practical design solution to the important problem in the field of air filtration. The results of this study can be used as a practical design guide to reduce pressure drop via depth filtration.

## 1. Introduction

Particulate matter (PM), consisting of solid particles, liquid aerosol, and gas and vapor compounds [1,2,3], is often classified by its aerodynamic diameter. PM 2.5, of which aerodynamic diameter ≤2.5 µm, is particularly reported as a serious threat to human health by the World Health Organization [4], because such an extremely small PM can be inhaled through respiratory organs, and then, circulated in the human body through blood vessels, potentially causing serious health problems [5,6,7,8,9,10].

Fiber-based filters have been widely used for separating PMs from contaminated air [11,12,13,14,15]. To manufacture conventional fiber-based filter media, the meltblown nonwoven process is typically employed using a polypropylene polymer [16]. In practical applications, high efficiency particulate air (HEPA) filters are used to provide maximum filtration performance for the most penetrating particle size, 0.1~0.5 µm count median diameter (CMD) [17,18,19]. While the conventional HEPA filters provide very high removal efficiency, at the filter design, it should be also considered that the pressure drop can increase quite steeply as the accumulated particles clog the pores of the filter [20,21,22]. As the filtration efficiency and pressure drop are often inversely correlated and trade off each other, the concept of quality factor (QF) is employed to calculate the relative filtration efficiency to a unit pressure drop [23,24]. In terms of QF, a higher value of QF is desirable as it means higher filtration efficiency, or lower particle penetration, at the unit pressure drop.

As a way to enhance filtration efficiency at a relatively low pressure drop, the charging process is commonly imposed on meltblown fibers [25,26,27,28]. As electrostatic attraction plays an additional role in capturing particles other than interception, impaction, diffusion, etc., the electrostatically charged (electret) filters generally provide better performance than the mechanical filters at the similar level of pressure drop [29,30]. Regardless of such efforts, pressure drop of filters tends to gradually increase at the constant airflow rate or air velocity, as the particles are loaded and collected on the filtration media [31,32]. The tendency of pressure drop increase is much greater for the solid particles than the liquid aerosol, and the pressure drop may be an important factor that limits the effective service life of filter materials, especially for the collection of solid particles. With the continued use of filters, particles are clogging up the pore of the filter membrane, which is the passage for air stream, and causing high resistance. High resistance or pressure drop of filter media is a critical issue for high-performing filters, as it could lead to the breathing resistance for filtering respirator users [33,34,35] and excessive energy consumption of filtration systems [36].

More specifically, the breathing resistance of respirator users may give negative physiological effects such as respiratory rate, heart rate, and blood pressure. The users may also experience thermal stress by the deteriorated air permeability; a study revealed that the air temperature anterior to the face increased about 7.5 °C [37]. In addition, workers wearing respirators, compared to workers without respirator wearing, showed lower work performance with higher oxygen consumption [38,39]. For the heating, ventilation, and air-conditioning (HVAC) system, more energy is consumed as the filter is getting clogged; as the air filter in the HVAC system collects dirt and particles, the filtering system would consume extra fan energy and cooling energy [40].

Considering that the filtering equipment used for human health can become a potential harm to health and environment, it is significant to design a high performing filter construction with a low pressure drop. A number of methods and ideas have been studied to decrease the filter resistance. For example, fibers with varied diameters were tested to examine the effect of fiber size on media porosity and resistance. Some studies reported that large fibers produced a high-quality factor media with low resistance [41,42]. Argumentatively, another study reported nanofibers in 60–100 μm can produce the “slip effect”, by which the frictional force between air molecules and fibers were reduced. The studies demonstrated the “slip effect” by the simulated work, and reported that the nanofibers in this size range are beneficial for decreasing the resistance [17,43,44,45]. However, the application of those studies may be limited because only the initial pressure drop has been considered. As the evolvement of pressure drop depends on various factors including pore size distribution and fibrous assembly, it is not sure that such submicron fibers would be still desirable for lowering the pressure drop during the prolonged filtration.

In the study that examined the effect of nonwoven bonding method on filter resistance, the air-through bonding that created porous 3D fibrous assembly was reported to give a superior quality factor to the thermal bonding method [42]. Likewise, a 3D media structure, in which microspheres and fibers were intermingled, displayed relatively lower resistance than the filter media constructed solely with fibers; in this study, the QF factor and the resistance was controlled by manipulating the size of microspheres and the ratio of microspheres to fibers, and demonstrated that microspheres played a role in separating the fibers, making a highly porous 3D composite membrane [46,47].

For the same amount of filter material, a number of split media in multi-layers were preferable to a single-layered, thicker media [48,49]. It was thought that even a slight space between the media could act as an air pathway, and this implies that the space between the layers could be utilized as a design factor to manipulate the performance and resistance. With such an assumption, this study aims at enhancing the particle loading performance with the lowered pressure drop and service life at a given set of filter combinations out of the following design factors: (1) number of filter layers, (2) web-to-web distance between filter layers, (3) use of spacer web between filter layers, and (4) face velocity. The main goal of this study is to suggest a practical design factor that is effective in lowering the pressure drop of filter construction by facilitating the depth loading. 

From the designed experiments of filter combinations, historical loading curves of penetration and pressure drop were analyzed to evaluate the clogging onset and the dust holding capacity. The solid-particle loading behavior was microscopically observed on each layer of multi-layered construction, to examine the load sharing of particles. Along with that, the numerical modeling and simulation were attempted to interpret the particle loading behavior, using the simplified fiber structures of tested filter. 

**Figure 1:**
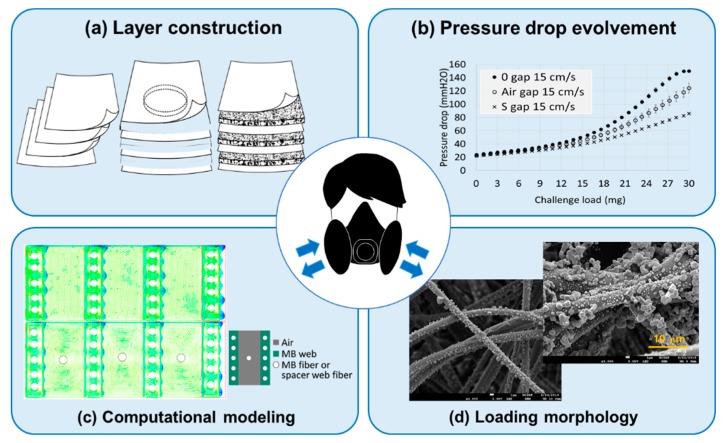
Schematic overview of study. (**a**) Filter layer construction was varied with multilayers of meltblown filter web, (**b**) Pressure drops of different layer-constructions were investigated, (**c**) Computational modeling was conducted to interpret the pressure drop behavior of different constructions, (**d**) Loading morphology of different constructions were investigated.

This study intends to provide a practical design solution to an important problem in the field of air filtration, employing both experimental investigation and numerical simulation in interpreting the data, which is a novel approach. The results of this study are anticipated to give a design insight to improve the quality factor with the lowered pressure drop, which will ultimately contribute to human health and environmental sustainability by enhancing the breathing comfort of respirator users and by reducing the energy consumption of the HVAC system. Figure 1 shows the schematic overview of this study.

## 2. Materials and Methods 

### 2.1. Materials

A conventional polypropylene (PP) meltblown (MB) electret was used as a filtration media in this study. A porous, fluffy web of polyethylene terephthalate (PET) from Aeropro Filter Industry (Shanghai) Co., Ltd. (Qingpu, Shanghai, China) was used as a spacer web (S), being inserted between the MB filter layers. An acrylic plate (5 mm thickness) with a circular hole of 71.4 mm was custom-made and used as a web holder for filtration test. This acrylic plate was also used to introduce a bulk air gap for multi-layered filter constructions, maintaining a 5 mm web-to-web distance. Sodium chloride (ACS grade, 99.5%) from Showa Chemical Industry Co., Ltd (Meguro-ku, Tokyo, Japan) was used to make a 2% NaCl aqueous solution and to generate a challenging particulate agent. The solidity and porosity of the webs were calculated using Equations (1) and (2) in the following [29]. The characteristics of webs are shown in Table 1:

Solidity (unitless) = *m*/(*A*⋅*t*⋅ρ), (1)

Porosity (%) = (1 − solidity) × 100 (%).(2)

*m* (g): sample mass

*A* (${\mathrm{cm}}^{2}$): sample area

*t* (mm): sample thickness

ρ (g/${\mathrm{cm}}^{3}$): polymer density (1.35 g/cm^3^ for PET, 0.91 g/cm^3^ for PP)

### 2.2. Preparation of Multi-Layer Construction

**Figure 2:**
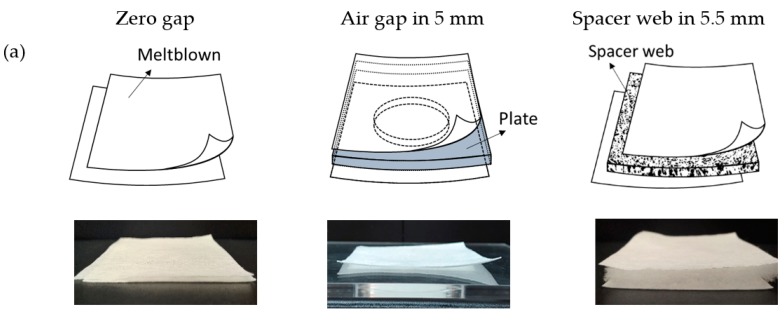

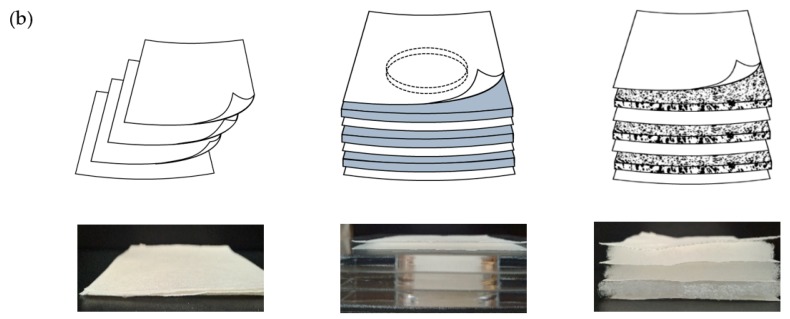
Experimental set-up for layer construction. (**a**) 2-Layer construction, (**b**) 4-Layer construction.

A multi-layered MB filtration media was constructed by varying the number of MB filter layers and web-to-web spacing, using either acrylic plates or spacer webs. Table 2 shows the test variables of this study. For the multi-layered constructions, the gaps between filter layers were adjusted to a zero gap, 5 mm air gap, or 5.5 mm spacer gap, by inserting 5 mm thick acrylic plates or 5.5 mm thick-spacer webs (S) between the filter webs, respectively. Different layer constructions tested in this study are illustrated in Figure 2. A single-layer MB filter web and a single-layer spacer web (S) were tested as controls, where S hardly showed filtration performance, with >99.5% penetration and < 0.5 mmH_2_O pressure drop. To examine the effect of air face velocity on pressure drop (dP), two different face velocities, 20 cm/s and 15 cm/s respectively, were employed. 

### 2.3. Filtration Performance

An automated filter tester (TSI 8130, TSI Inc., Shoreview, MN, USA) was used to evaluate the filtration performance test with NaCl particles in the most penetrating particle size range (count median diameter of about 0.075 ± 0.02 µm), of which the equilibrium charges are neutralized. The NaCl aerosol with mass concentration of 25 ± 0.2 g/L was passed through the sample area of 40 cm^2^ at the face velocity of 15 cm/s (36 lpm) and 20 cm/s (48 lpm), respectively. The NaCl particles were continuously challenged to the filter media to a total mass of 30 mg. As indicators of filtration performance, % penetration (Pn) of aerosol and pressure drop (dP) were monitored throughout the particle loading. The quality factor (QF) was calculated following Equation (3) to evaluate the relative filtration efficiency at a unit pressure drop [50]. The highest measurable limit for dP by this filter tester was 150 mm H_2_O, thus after dP reached 150 mm H_2_O, dP was recorded as the same value:

(3) $$\mathrm{Quality}\;\mathrm{factor}\;({\mathrm{mm}\;H}_{2}O^{-1})\;=\;-\ln\left(\frac{\%\;\mathrm{penetration}/100\%}{\mathrm{pressure}\;\mathrm{drop}\;\left({\mathrm{mm}\;H}_{2}O\right)}\right).$$

### 2.4. Morphological Characterization

Morphological characterization of filter layers with and without NaCl loading (up to 30 mg) was performed using a field emission scanning electron microscope (FE-SEM, JSM-7800F, JEOL Ltd, Akishima, Tokyo, Japan). Prior to FE-SEM analysis, a Pt coating on fibrous samples were done for 120 s at 20 mA, using a sputter coater (108auto, Cressington Scientific Inc., Watford, Hertfordshire, UK). The fiber diameter of MB and S webs were measured by selecting 20 random fibers from SEM images.

### 2.5. Modeling and Simulation

**Figure 3:**
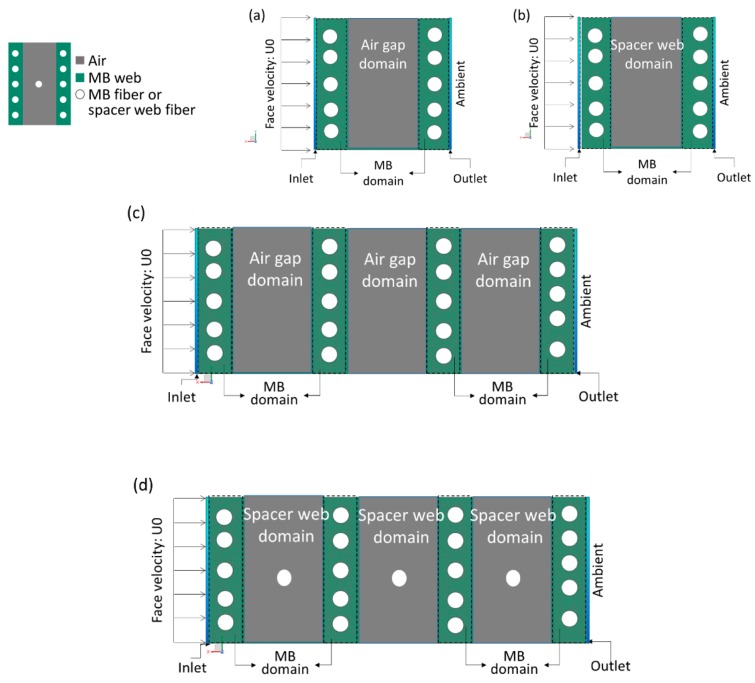
Developed computational fluid dynamics models of given filter constructions. (**a**) 2 MB layers and 1 air gap, (**b**) 2 MB layers and 1 space web, (**c**) 4 MB layers and 3 air gaps, (**d**) 4 MB layers and 3 spacer webs.

In order to analyze flow behaviors in the considered filter constructions of this study, computational fluid dynamics (CFD) simulation was carried out via the commercial solver of Simcenter Flow in Siemens® NX. From the experimental test, since different loading performance was observed for 2 MB layers and 4 MB layers with air gap and spacer web combinations, 4 simulation models of 2 MB layers with 1 air gap, 2 MB layers with 1 space web, 4 MB layers with 3 air gaps, and 4 MB layers with 3 spacer webs were developed as shown in Figure 3. Relative shapes and sizes of MB layers, spacer webs, and fibers in Figure 3 were decided from the characteristic data of chosen nonwoven materials shown in Table 1. The relative comparisons between MB/air gap and MB/spacer web models were made to mainly interpret the flow velocity patterns, which affect the particle loading performance along with service life. The higher velocity was corresponded with the higher pressure drop in the interpretation. As the flow patterns can be dominantly influenced by the porous domain, in this model, the relative porosities in the spacer web and the MB web were adjusted, keeping the fiber diameters for MB and S web the same. To simplify the air flow simulation, the interactions between the air and NaCl test particles were ignored in this model. Flow of air was analyzed employing the standard k-ε model. Inlet velocity of U0 was set to 15 cm/s, and the outlet was given as an ambient condition for all analysis models.

## 3. Results and Discussions

### 3.1. Effect of Layer Construction on Filtration Performance

The initial penetration (In. Pn) of a single MB web (at 15 cm/s) was 3.62%; when the MB was layered, the dual layer (2L-0 gap) showed 0.12% penetration, which approximately corresponds to the theoretical penetration value (3.62% × 3.62% = 0.13%). The experimental value of maximum penetration of dual layer (0.83%) was lower than the predicted value (11.77% × 11.77% = 1.38%). The experimental value of initial dP for 2-layers (2L-0 gap) (12.1 mm H_2_O) was lower than the predicted value (6.9 mm H_2_O × 2 = 13.8 mm H_2_O), and the tendency of lower pressure drop than the predicted was more apparent for 4-layered (4L) structures (24.0 mm H_2_O for 4L-0 gap at 15 cm/s). The results demonstrate that a multi-layered design is beneficial in lowering the dP, compared to a single layer of bulk material in the same mass. 

**Figure 4:**
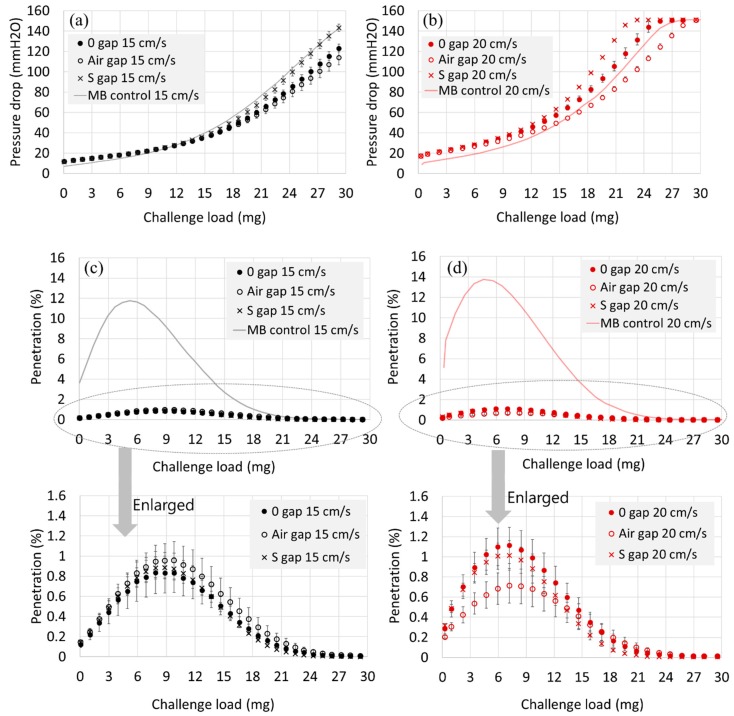
Pressure drop and penetration development for 2-layer constructions during 30 mg of NaCl loading. (**a**) Pressure drop at 15 cm/s, (**b**) Pressure drop at 20 cm/s, (**c**) Penetration at 15 cm/s, (**d**) Penetration at 20 cm/s. Note. For a single layer of spacer web, Pn (%) remained >99.5% and dP (mm H_2_O) remained <0.5 mm H_2_O throughout the loading.

From Figure 4, when air gap or spacer web was inserted for the 2 MB-layer structure, the initial dP was hardly affected. However, as the accumulated particle loading increased, the dP evolved more slowly when the air gap was inserted between the filter layers. While the air gap between the layers acted as the effective flow channel, the S web did not help reduce the pressure drop. On the contrary, the spacer web adversely affected the dP; as the pressure drop (dP) of S web itself is negligible, it was speculated that the increased dP was due to the inhibited air flow by the S web. In the edge of samples 2L-S gap, the S webs and the MB webs were squeezed together, and the air flow at the edges could be hindered, reducing the effective surface area for air passage. 

**Figure 5:**
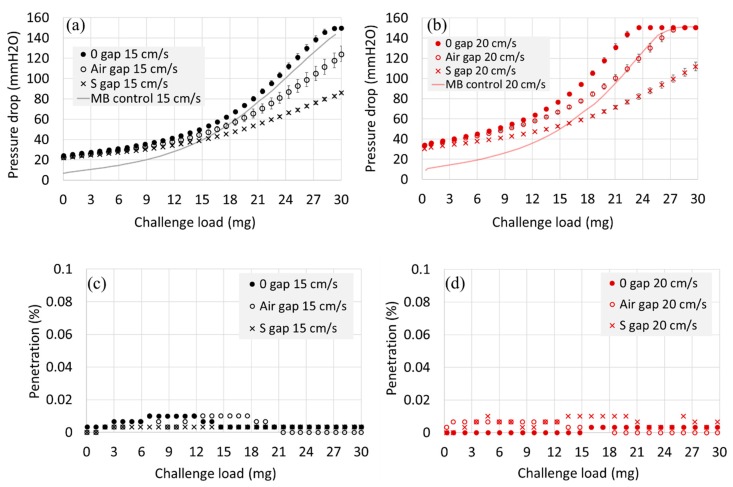
Pressure drop and penetration development for 4-layer constructions during 30 mg of NaCl loading. (**a**) Pressure drop for 15 cm/s, (**b**) Pressure drop for 20 cm/s, (**c**) Penetration for 15 cm/s, (**d**) Penetration for 20 cm/s. Note. For a single layer of spacer web, Pn (%) remained >99.5% and dP (mm H_2_O) remained <0.5 mm H_2_O throughout the loading.

For the 4L construction, either the air gap or the spacer web insertion (S-gap) was beneficial in reducing the dP, and the effect was greater when the face velocity was increased (Figure 5). That is, at the face velocity of 15 cm/s, the dP at 20 mg loading was reduced by 17.6% and by 32.5% for the 4L-air gap and for the 4L-S gap, respectively. At 20 cm/s, the dP at 20 mg was reduced by 21.9% and by 43.1% for 4L-air gap and for 4L-S gap, respectively. The dP reduction of 17%~43% could be significant in practical applications for respirator users. It is noteworthy that for 4-layer constructions, the use of spacer web between the filter layers was more effective in reducing the dP than the use of bulk air gap, where the spacer web acted as a direct air flow channel, effectively distributing the air flow and dP. 

### 3.2. Depth Filtration and Loading Capacity

Generally, the build-up of pressure drop is much faster for the solid aerosol than the liquid aerosol, because the pores of a filter are easily clogged by the dendrite formation of solid particles. On the other hand, liquid aerosol spreads on the filter fibers instead of clogging the pores, and the build-up of dP is insignificant or much slower for liquid aerosol. For a porous electret filter, as solid particles are captured on the fiber surface, the surface charges are masked by the captured particles; as a result, the filter performance is deteriorated and the penetration increases, until it reaches the maximum penetration (Max. Pn). With the continued accumulation of solid particles after reaching the Max. Pn, the clogging finally occurs, decreasing the penetration. As the penetration decreases, the dP of filter develops steeply from the clogging point. In this study, it was assumed that the challenged loads (mg) at the Max. Pn is the clogging onset. 

For 2-layer (2L) constructions, the clogging was delayed compared to the single-layer (1L) media. The clogging onset of 2-layer construction (9.8 mg of NaCl loading) was a little less than two times of the single-layer clogging onset (5.8 mg of NaCl loading) (Table 3). For 4-layer (4L) constructions, the clogging onset for 4-layer was not clearly observed, as the penetration during the loading remained almost equally low (≤0.01%). For 2-layer structures, the Max. Pn, or the clogging onset appeared at the same amount of NaCl loading, regardless of the different gap constructions; the clogging points were observed at 9.8 mg and 7.2 mg of challenged mass, respectively, for the face velocities of 15 and 20 cm/s. As the face velocity increases, not only the pressure drop increased, but also occurred the clogging more quickly.

**Figure 6:**
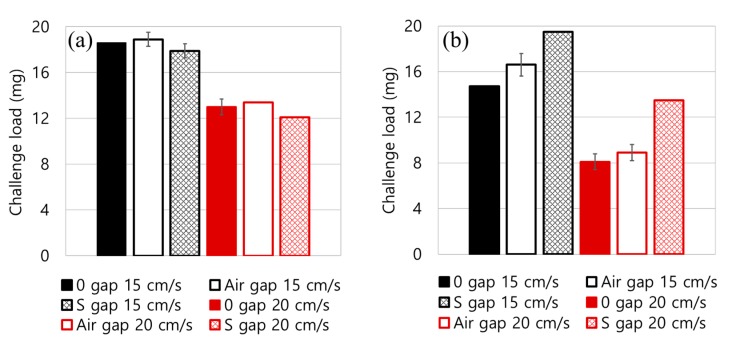
Service life associated with the pressure drop for different layer constructions. (**a**) Challenged load at 50 mm H_2_O for 2-layer construction, (**b**) Challenged load at 50 mm H_2_O for 4-layer construction.

Usually, for the solid particles, the service life of the filtering product is limited by the build-up of dP. To compare the pressure drop-based service life for different filter constructions, the challenged NaCl mass that reaches an arbitrary pressure drop of 50 mm H_2_O was also examined for differently constructed media (Table 3 and Figure 6). For 2-layer constructions, the insertion of air gap or spacer (S) web hardly changed the loading mass to reach 50 mm H_2_O. For the 4-layer constructions, the insertion of air gap or spacer web increased the challenged mass to reach 50 mm H_2_O, increasing the loading capacity or the effective service life in terms of dP. 

**Figure 7:**
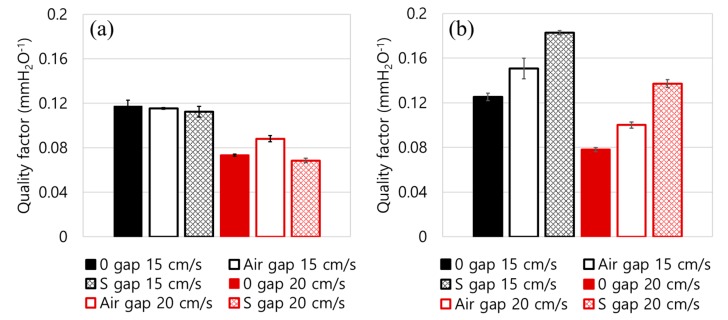
Quality factor at 20 mg NaCl loading. (**a**) Quality factor for 2-layer construction, (**b**) Quality factor for 4-layer construction.

While both the air gap and the S web allowed the air pathway between the compact MB layers, the effect of S web on the increased service life was greater than that of the air gap. This implicates that the spacer web, which is comprised of porous, fluffy fibers, distributes the air flow more effectively than the bulk of air gap. The fluffy fibers constitute large pores that act as tortuous multi-channels for directing the air flow, so that the dP is well distributed to the entire surface area. This also led to the depth loading, in contrast to surface loading, where the particles were effectively collected through multi-layers, sharing the particle mass. The well-distributed dP for the 4L-S gap construction led to the extended service life as shown in Figure 5 and Figure 6. On the other hand, the bulk air gap provides a free space between the MB layers, causing preferential air flow through the gaps, which results in a poor distribution of air flow and the pressure drop [49]. Though the insertion of air gap or S-gap considerably reduced the dP, the penetrations were very similar for the different constructions. As a result, the quality factor of the 4L with the spacer gap was considerably higher than the other constructions (Figure 7). 

### 3.3. Morphological Characterization

**Figure 8:**
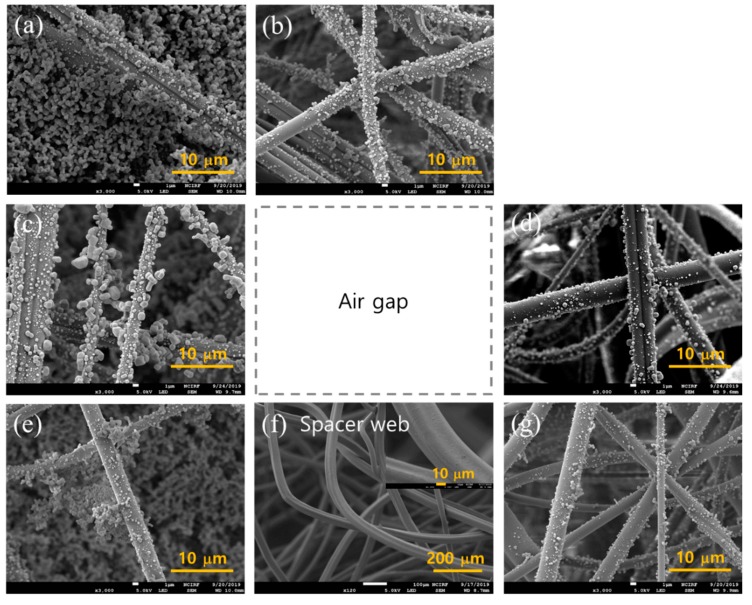
SEM images of 2-layer construction after 30 mg loading (15 cm/s). (**a**) 1st MB layer with 0 gap, (**b**) 2nd MB layer with 0 gap, (**c**) 1st MB layer with air gap, (**d**) 2nd MB layer with air gap, (**e**) 1st MB layer with S gap, (**f**) S web inserted between MB layers, (**g**) 2nd MB layer with S gap.

The morphology of particle loading was observed when 30 mg of NaCl was challenged to the layer constructions. For 2-layer constructions in Figure 8, there were no observable differences among the different constructions, showing that the first upper most layer had the most of particles loaded. The dP of 2-layer constructions with and without the gap were not much different. Likewise, the clogging onset and the effective service life of all 2-layer constructions were similar. From Figure 8, the clogging seemed to occur at the top most layer, and after 30 mg of NaCl loading, the particle cakes were formed similarly, regardless of construction types. The spacer web did not have any particles loaded on the surface, demonstrating that the S web had negligible contribution to the particle capture. The S web contributed solely as a channeled-separator or air pathways. 

**Figure 9:**
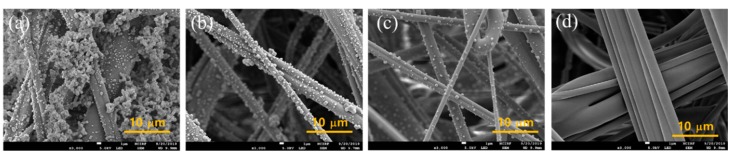

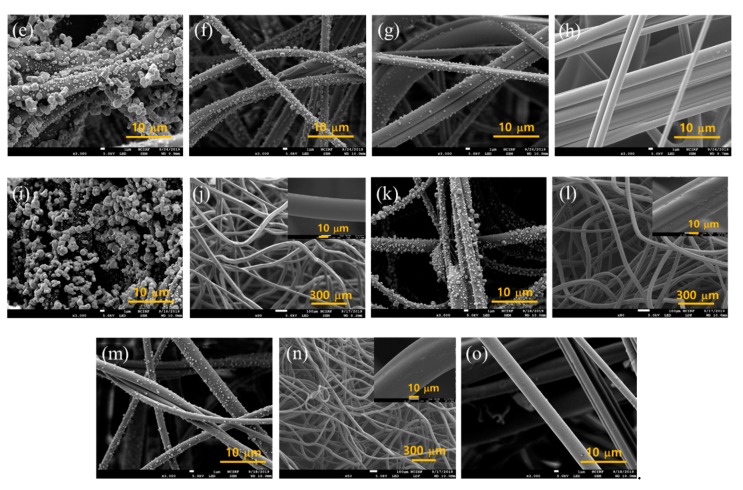
SEM images of 4-Layer construction after 30 mg loading (15 cm/s). (**a**–**d**), 4-layers without gap; (**a**) 1st MB layer with 0 gap, (**b**) 2nd MB layer with 0 gap, (**c**) 3rd MB layer with 0 gap, (**d**) 4th MB layer with 0 gap. (**e**)~(**h**), 4-layers with 5 mm air gap; (**e**) 1st MB layer with air gap, (**f**) 2nd MB layer with air gap, (**g**) 3rd MB layer with air gap, (**h**) 4th MB layer with air gap. (**i**–**o**), 4-MB layers with spacer webs; (**i**) 1st MB layer with S gap, (**j**) 1st S layer, (**k**) 2nd MB layer with S gap, (**l**) 2nd S layer, (**m**) 3rd MB layer with S gap, (**n**) 3rd S layer, (**o**) 4th MB layer with S gap.

In Figure 9, the 4-layer constructions with and without the gap are shown. The top most layer in the 4L-0 gap (Figure 9a) showed the most particle cakes compared to that in the 4L-air gap (Figure 9e) or the 4L-S gap (Figure 9i). The 4th layer at the bottom (Figure 9d,h,o) showed almost no particles, showing that 3 layers may be sufficient to capture most particles in 30 mg mass loading. It is noteworthy that the 2nd layers (Figure 9b,f,k) show slightly different levels of particle loading; the 2nd layer of the 4L-S gap appeared to show more particles loaded on the fiber surface, implying that the load sharing of NaCl particles was rather efficient for this construction (4L-S gap). The NaCl mass loaded on each layer of 4-layer constructions, after 30 mg challenging, was measured in Table 4. In the 4L-S gap, the loaded NaCl particles were shared from the 1st, 2nd, and 3rd MB layers with 87.7%, 9%, 3.3%, respectively. In contrast, the constructions of the 4L-0 gap and the 4L-air gap had NaCl mass shared only on the 1st and 2nd MB layers; with the very marginal share on the 2nd layer. From the result, it was confirmed that the 4L-S gap had the most effective load share of solid particles, via the uniform air flow distribution. The effective load share is the evidence of depth filtration, which eventually improves the load capacity and service life.

The results of this study give practical insight on designing filter products. Not only the presence of the air gap but also, or more importantly, the type of air channel between the filter layers makes a significant difference in lowering the pressure drop and extending the service life, which would be translated as the breathing comfort for filtering respirator users or energy saving for the HVAC filter system. The physiological convenience and energy saving benefit can be important decision factors when choosing a product, considering sustainable use or consumption. The results indicate that the proper design of air channels in a filter product would be an effective way of reducing the dP and extending the service life. The use of fluffy web as a spacer could be a convenient method to implement the air channels, without needing additional spacer frames. 

### 3.4. Modeling and Simulation

**Figure 10:**
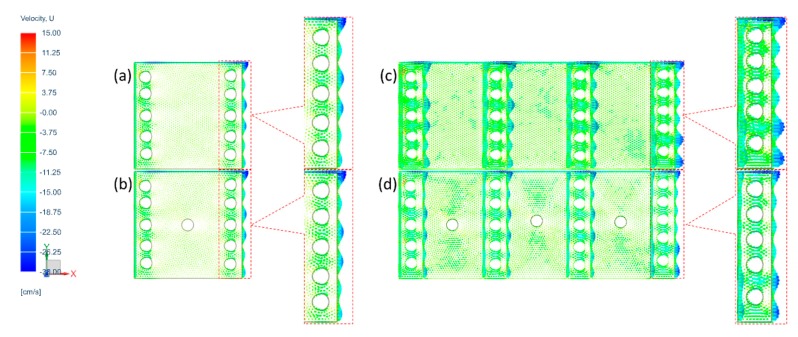
Velocity profile of (**a**) 2L- air gap, (**b**) 2L-S gap, (c**)** 4L-air gap, (**d**) 4L-S gap. The inlet velocity was set as 15 cm/s, and the outlet velocity was simulated.

Since the experimental results in general were affected by the flow behavior around the outlet section of test set-up, it is important to analyze the velocity behavior. In this regard, the contour plot of the velocity profile was compared for different constructions at the developed analysis models, using the inlet velocity of 15 cm/s. As shown in Figure 10a,b, the two models with the 2L-air gap and 2L-S gap have similar velocity patterns at the outlet section, although the velocity at the center of the 2L-S gap displays a different pattern. It appears that 2-layer constructions could not provide sufficient room to hold particles inside, thus particles are stacked on top of the very first layer of MB, forming a stable cake, as is commonly observed at the surface filtration process.

When the 4L-air gap and 4L-S gap were analyzed (Figure 10c,d), overall velocity of the 4L-S gap was more uniformly distributed at the outlet section than the 4L-air gap. As the velocity gets uniform in the filter media, there are more chances to collect dust particles during the filtration process, and thus, the filtration efficiency generally increases. This simulation result demonstrates that the filter media with the spacer webs have higher particle holding capacity, with effective load share, which allows depth filter instead of surface filtration. This would eventually improve the service life of filters, delaying the clogging. In this notion, the flow simulation results are well matched with the experimental results.

## 4. Conclusions

This study aimed at defining a practical design factor that is effective in lowering the pressure drop and enhancing the particle loading performance at the given set of filter materials, applying the gap design between the filter webs. The web-to-web space was implemented either maintaining a bulk air gap with 5 mm distance or inserting a porous spacer web with 5.5 mm thickness. The effect of web-to-web distance was apparent for 4-layer constructions, and the effect was greater at higher face velocity. For the 4-layer constructions, both the air gap and the spacer web were beneficial in reducing the pressure drop. The implementation of the spacer web in 4-layer construction was more effective in reducing the dP than that of air gap, demonstrating the porous spacer web acted as air channels that enabled effective distribution of pressure drop between the compact MB layers. 

The clogging was delayed for the multi-layered constructions, compared to the single-layer filter. As the face velocity increased, not only did the pressure drop increase, but the clogging also occurred more quickly. To compare the pressure drop-based service life of different filter constructions, the challenged NaCl mass that reached the pressure drop of 50 mm H_2_O was examined. While 2-layer construction did not show an apparent tendency of dP with the gap insertion, 4L construction displayed a strong tendency of lowered dP and extended service life with the introduction of bulk air gap or the spacer web. Also, the insertion of air gap or spacer web increased the loading capacity or the effective service life. From the SEM images, the 2nd layer of the 4L-S gap showed more particle loading than the 2nd layers in other constructions, implying that in the 4L-S gap, the load sharing between the top and the 2nd layer was rather efficiently made. The measurement of mass loading for each layer of 4-layer construction supports this observation. 

Flow simulation to interpret the experimental results was carried out while comparing flow velocity behaviors at the given filter media constructions. From the simulation, two filtration behaviors of surface filtration and depth filtration were observed depending on the filter media constructions. The simulation results were well aligned with the obtained experimental results.

The result of this study provides practical insight on designing filter products. Not only the presence of air space, but also the type of spacing between the filter layers made a significant difference in reducing the pressure drop and loading capacity. This study intends to provide a practical design solution to an important problem in the field of air filtration, employing both experimental investigation and numerical simulation in interpreting the data, which is a novel approach. The results of this study are anticipated to give design insight to improve the depth filtration with lowered pressure drop, which will ultimately contribute to human health and environmental sustainability by enhancing the breathing comfort of respirator users and by reducing the energy consumption of the HVAC system. While certain aspects of the media construction in this study may also apply to the pressure drop of liquid filters, the scope of this study remains limited to air filtration, such as air purifying respirators and the HVAC system, because the filtration mechanisms between the air filter and liquid filter are quite different.

## Figures and Tables

**Table 1:** Characteristics of nonwoven materials.

	Meltblown Filter Web (MB)	Spacer Web (S)
Material	Polypropylene	Polyethylene terephthalate
Basis weight (g/m^2^)	34 (±3, n = 5)	76 (±5, n = 5)
Thickness (mm)	0.28 (±0.03, n = 5)	5.5 (±0.2, n = 5)
Solidity (unitless)	0.133	0.010
Porosity (%)	86.7	99.0
Mean fiber diameter (μm)	2.6 (±1.1, n = 20)	35.0 (±4.3, n = 20)

Note: Inserts in parenthesis are standard deviations of measurements and the number of samples measured.

**Table 2:** Test variables.

		0 mm Gap		Air Gap (5 mm)		Spacer Web (5.5 mm)	
Face velocity (cm/s)		15	20	15	20	15	20
No. MB layers	1-Layer (control)	O	O	-	-	-	-
	2-Layer	O	O	O	O	O	O
	4-Layer	O	O	O	O	O	O

**Table 3:** Summary of loading performance of different filter constructions.

Facevel.	Constr.	In. pn (%)	Max. pn (%)	In. dP (mm H_2_O)	dP at max. pn (mm H_2_O)	Challenge Load at max. pn (mg)	Challenge at 50 mmH_2_O (mg)	dP at 20 mg Challenge (mm H_2_O)
15 cm/s	1L-MB	3.62	11.77	6.9	13.5	5.2	17.0	69.2
	2L-0 gap	0.12	0.83	12.1	24.0	9.8	18.6	57.7
	2L-air gap	0.14	0.95	11.9	23.9	9.8	18.8	55.4
	2L-S gap	0.14	0.89	11.8	24.0	9.8	17.9	64.0
	4L-0 gap	≤0.01	≤0.01	24.2	N.D.	N.D.	14.7	76.9
	4L-air gap	≤0.01	≤0.01	22.7	N.D.	N.D.	16.6	63.4
	4L-S gap	≤0.01	≤0.01	22.0	N.D.	N.D.	19.5	51.9
20 cm/s	1L-MB	5.2	13.77	9.1	16.8	3.71	11.6	88.2
	2L-0 gap	0.3	1.11	17.6	31.3	7.2	13.0	99.7
	2L-air gap	0.2	0.71	17.5	29.4	7.2	13.4	79.1
	2L-S gap	0.3	1.01	17.5	31.6	7.2	12.1	112.9
	4L-0 gap	≤0.01	≤0.01	34.1	N.D.	N.D.	8.1	118.0
	4L-air gap	≤0.01	≤0.01	33.2	N.D.	N.D.	8.9	92.2
	4L-S gap	≤0.01	≤0.01	30.4	N.D.	N.D.	13.5	67.2

Note: For 4-layer constructions, the penetration was ≤0.01%, and the pressure drop at the maximum penetration was not clearly detected (N.D.).

**Table 4:** Loaded mass for each layer for 4-layer constructions.

Gap	0 mm Gap		Air Gap		S Gap	
Layer Tested	Mass Loaded (mg)	% Load Share (%)	Mass Loaded (mg)	% Load Share (%)	Mass Loaded (mg)	% Load Share (%)
1st layer MB	29.3 (±0.6)	97.8 (±1.9)	28.3 (±1.2)	96.6 (±0.1)	25.7 (±0.6)	85.6 (±1.9)
2nd layer MB	0.7 (±0.6)	2.2 (±1.9)	1.3 (±0.6)	4.5 (±1.9)	2.7 (±0)	8.9 (±1.9)
3rd layer MB	-	-	-	-	1 (±0)	3.3 (±0)
4th layer MB	-	-	-	-	-	-

Note: The lowest detection limit of balance was 1 mg. The lower mass was not measurable.
