# Erratum: Design of Web-to-Web Spacing for the Reduced Pressure Drop and Effective Depth Filtration. *Polymers* 2020, *11*, 1882

**DOI:** 10.3390/polym12040938

**Published:** 2020-04-17

**Authors:** Sanghyun Roh, Kangsoo Park, Jooyoun Kim

**Affiliations:** 1 Department of Textiles, Merchandising and Fashion Design, Seoul National University, Seoul 08826, Korea; shtkgus011@snu.ac.kr; 2 R&D Center, Satrec Initiative Co., Ltd., Daejeon 34054, Korea; mepgsoo@gmail.com; 3 Research Institute of Human Ecology, Seoul National University, Seoul 08826, Korea

The authors wish to make a change to the published paper [1]. In the original manuscript, there is a mistake in the Equation (3). The corrected Equation (3) is presented below.

(3) $$\mathrm{Quality}\;\mathrm{factor}\;(\mathrm{mm}\;H_{2}O^{-1})=\frac{\;-\ln(\%\;\mathrm{penetration}/100\%)}{\mathrm{pressure}\;\mathrm{drop}\;\left({\mathrm{mm}\;H}_{2}O\right)}$$

The authors apologize for any inconvenience caused, and this change does not affect the scientific results. The manuscript will be updated, and the original will remain online on the article webpage at https://www.mdpi.com/2073-4360/11/11/1822.
